# Isolation and Properties of Cellulose Nanofibrils from *Coconut Palm Petioles* by Different Mechanical Process

**DOI:** 10.1371/journal.pone.0122123

**Published:** 2015-04-14

**Authors:** Changyan Xu, Sailing Zhu, Cheng Xing, Dagang Li, Nanfeng Zhu, Handong Zhou

**Affiliations:** 1 Packaging Engineering Department, Nanjing Forestry University, Nanjing, Jiangsu, China; 2 School of Packaging, Michigan State University, East Lansing, Michigan, United States of America; Michigan Technological University, UNITED STATES

## Abstract

In this study, cellulose nanofibrils (CNFs) were successfully isolated from coconut palm petiole residues falling off naturally with chemical pretreatments and mechanical treatments by a grinder and a homogenizor. FTIR spectra analysis showed that most of hemicellulose and lignin were removed from the fiber after chemical pretreatments. The compositions of CNFS indicated that high purity of nanofibrils with cellulose contain more than 95% was obtained. X-ray diffractogram demonstrated that chemical pretreatments significantly increased the crystallinity of CNFs from 38.00% to 70.36%; however, 10-15 times of grinding operation followed by homogenizing treatment after the chemical pretreatments did not significantly improve the crystallinity of CNFs. On the contrary, further grinding operation could destroy crystalline regions of the cellulose. SEM image indicated that high quality of CNFs could be isolated from coconut palm petiole residues with chemical treatments in combination of 15 times of grinding followed by 10 times of homogenization and the aspect ratio of the obtained CNFs ranged from 320 to 640. The result of TGA-DTG revealed that the chemical-mechanical treatments improved thermal stability of fiber samples, and the CNFs with 15 grinding passing times had the best thermal stability. This work suggests that the CNFs can be successfully extracted from coconut palm petiole residues and it may be a potential feedstock for nanofiber reinforced composites due to its high aspect ratio and crystallinity.

## Instruction

*Coconut palm* (*Cocosnucifera L.*) is one of the most important agro-industry plantsin tropical and sub-tropical regions.The continuously increasedcoconut residues cause a serious environmental problem in China. With the increase of environmental awareness, more and more attention has been paid to the topic of how to utilize those coconut residues effectively for value-added and environmentally friendly products. The coconut residues are, by their very nature,a lignocellulosic material with renewable, biodegradable andbiocompatible characteristics, and should be an ideal raw material resource for natural fibers [1,2]. As early as in 1982, Satyanarayana et al. examined the properties of rachis, rachilla, spathe, leaf sheath and petiole bark of coconut palm tree (*Cocosnucifera*, *linn.*), and concluded that the fibers coming from different parts of the tree could be used for various applications, especially in composite processing because of the physical and mechanical properties exhibited. Thereafter, some reports have verified the opinion that coconut fibers are suitable as a reinforcing materialin polymer-matrix composites. Monteiro et al. obtained coconut coir fiber from disregarded coconut shells and prepared coir fiber-polyester composites with amounts of coir fiber up to 50 wt% [3].Bledzkialso confirmed the potential of coconut shellas an alternative or together with wood fiberfor thermoplastic composites and successfully fabricated polypropylene composites with 40 wt% of fiber loading [4].

In the recent decades, cellulose has been one of the novel materials being developed from various natural resources [5]. Generally a cellulose chain is a polydisperselinear polymer of poly-*β(1*,*4)-D*-glucose residues and the degree of polymerization of wood and cotton cellulose chains is approximately 10,000–15,000 [6].In cell walls, cellulose micro-fibril bundles exist encased by embedding matrix such as hemicellulose and lignin, and the cellulose molecular chains form fibrous structural micro-fibrils, which are 3–4nm wide[7,8]. It is necessary for cellulose micro and nano-fibril fibrillation process to remove such matrix substances. The multi-layered structure of the cell walls with hemicellulose and lignin as a matrix between the micro-fibrils requires some chemical and mechanical treatments to extract the cellulose micro/nano-fibril from the cell wall. Many efforts have been made to develop adequate and commercially viable processes for disintegrating cellulose fibers into their structural components at a nanoscale[9]. The chemical pre-treatment includes a series of chemical treatments to remove most of wax, extractives, hemicelluloses and lignin of fibres, and then mechanically breaks down the fibres to micro/nanoscale fibril by grinding, ultra-sonication, highpressure homogenization or a combination of those processing methods.

In fact, nano-cellulose was successfully isolated from soy hulls[10], pineapple[11], corncob[12], cassava baggase[13], hemp fibres[14], rice husk[15], and sludge[16]. As a member of the agricultural residue family, coconut waste has attracted much attention asraw materials of cellulose fibers. Maheswari et al. extracted cellulose microfibrils with diameters in the range of 10–15 mm in length from coconut palm leaf sheath using chlorination and alkaline extraction process. Rosa et al. also isolated ultrathin cellulose nanowhiskers from coconut husk fibers with diameters as low as 5 nm and the aspect ratio was up to 60 by a method of combination of pre-delignification process and sulfuric acid hydrolysis treatments [17].However, there are very few reports in literature about utilization of coconut palm petiole residues as a raw material of cellulose micro/nano fibrils. Therefore, the aim of this study is to isolate and characterize cellulose nano-fibrils from coconut palm petiole residues falling off naturally with chemical pretreatments followed bythe mechanical treatment. The chemical pretreatments used in this study involved benzene/ethanol treatment, chlorite delignification, alkaline extraction as well as hydrochloric acid processing, and the mechanical treatment consisted of grinding through a grinder with 10–20 passing times and passing througha homogenizer 10 times. FTIR was used to collect information of functional group changing of the fibers after each chemical treatment. Crystallinity and crystal type of the coconut palm petiole fibers before and after treatments were investigated by X-ray diffraction.The morphology of the obtained CNFs and the degradation characteristics of coconut palm petiole fibers before and after chemical-mechanical treatment were also evaluated by SEM and TGA, respectively.

## Experimental

### Materials and Methods

Coconut palm petiole woody powder,provided byHainan Kunlun New Material Science & Technology Co.,Ltd.as source of cellulose in this study was cleaned with water, air dried, broken to the size of 2–3 mm × 6–7 mm with a L-905 shredder, grinded into powders with a FZ102 miniature plants grinder (TAISITE instrument Co., Tianjin), and sieved under 100 mesh (Zhang Xing Sand Screen Factory, Zhejiang province).Chemical composition of the coconut palm petiole powder was preliminarily investigated as showing in Table 1 [18]. Ethanol,toluene, hydrochloric acid, acidified sodium chlorite and sodium hydroxide pellets were purchased from Nanjing Chemical Reagent Co., Ltd. and all reagents were analytical grade.

**Table 1:** Physical properties of coconut palm petiole fibers.

Component	Content/wt%
Cellulose	33.29±0.09
Hemicellulose	33.61±0.07
Lignin	19.87±0.08
Alcohol benzene extractive	1.27±0.05
Ash	5.5±0.05

Note: The data after symbol ± were Stdev.

### Preparation of CNFs

**Fig 1:**
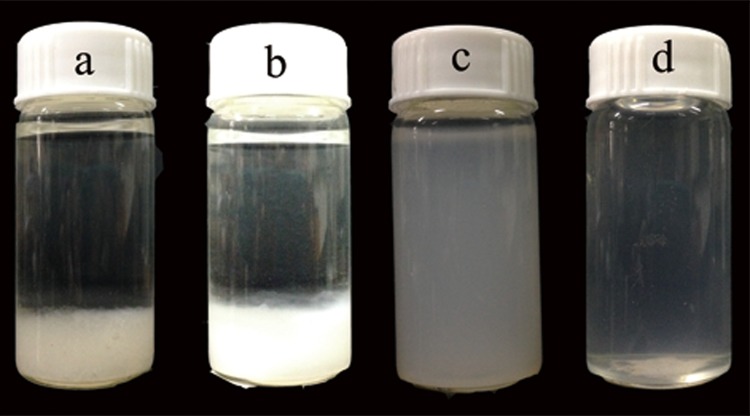
Photographs of samples in different stages: (a) after acidified sodium chlorite treatment, (b) after sodium hydroxide and hydrochloric acid treatment, (c) after grinding, and (d) after homogenization.

The coconut palm petiole powder(10g)was treated in mixture of benzene/ethanol (2:1 by volume) for 6 hours in a Soxhletapparatus(SXT-06, Shanghai Hongji Instrument LO., Ltd.) to remove the wax and other extractives [19]. Then, the sample was delignificated with 1 wt%acidified sodium chlorite water solutionat 75°C for 1 hour, and repeated this operation 3 times. In order to remove hemicellulose,residual starch and pectin, the resulted sample as shown in Fig 1a was filtered and washed with distilled water and further treated in 1.9wt%sodium hydroxide at 90°C for 2 hours. Afterbeing filtered and washed withdistilled water to a neutral pH value, the sample was treated with2.7wt%hydrochloric acid solution at 80°C for 2 hours, and then filtered and washed withdistilled water to neutral. The chemical pre-treated sample as shown in Fig 1b was then mechanically ground10, 15 and 20 times respectively with a MKCA6-3 grinder (Masuko Corp., Japan) at 1,500 rpmThe grinded sample as shown in Fig 1c was diluted to 0.6 wt% with distilled water and passed through a homogenizer10 times (EmulsiFlex-C3, AVESTIN, Inc., Canada) at pressure of 1000 Bar. The resulted final cellulose suspension as shown in Fig 1d was frozen at -18°C for 12 hours with a DW-FL200A (Meiling Cryogenic Technology Co., Ltd., China), followed by being frozen-drying for 48 hours at -45°C by Xianou-10 (Xianqu Biological Technology Co., Ltd., China), resulting in freeze-dried CNFs. Here, the CNFs samples with 10, 15 and 20 passing times of a grinder weredesignated in CNF-1, CNF-2 and CNF-3, respectively.

## Characterization

### Chemical composition

Chemical composition of the obtained CNFs was determined according to the methods reported by the Technical Association of Pulp and Paper Industry (TAPPI). Holocellulose content of the sample was determined by treating it with mixture solution of NaClO_3_ and NaOH [20]. After further treating the above sample with NaOH, α-cellulose content was obtained. Hemicellulose content was the disparity between the values of holocellulose and α-cellulose. The average values were calculated from the data of three samples.

### Fourier Transform-infra Red Spectroscope (FTIR) analysis

The untreated, benzene-ethanoltreated,acidified sodium chloritetreated and sodium hydroxidetreated coconut palm petiole fiber samples were analyzed with FTIR to collect detailed information of functional groups after each chemicaltreatment.FTIR analysis wasrecorded using a FTIRspectrometer (Nicolet IS10, Thermo scientific, America)under an attenuated total reflectancemode.Allspectra were obtained by accumulation of 64 scans, withresolution of 2 cm^-1^ at 500–4000 cm^-1^.

### X-ray Diffraction (XRD) analysis

XRD analysisof the samples was carried out with a diffractometer (Ultima IV) at power of 40KV, current of 30mA and CuKα radiation. X-ray diffractograms were obtained atroom temperature within a 2θranging from 5° to 40° with a scan rateof 2°min^-1^. The crystallinity index (Icr) of the sample was determined with the equation as follows [21],

$$I_{cr}=\frac{{}_{I_{002}}{\_}_{I_{am}}}{{}_{I_{002}}}\times 100\%$$ (1)

Where Icrexpresses the relative degree of crystallinity, I_002_ is themaximum intensity of the (002) lattice diffraction at 2θ = 22°, andI_am_ is the intensity of diffraction at 2θ = 18°. I_002_ represents bothcrystalline and amorphous regions while I_am_ represents only theamorphous part [5,12,15].

### Field Emission Scanning Electron Microscope (FE-SEM) study

After being coated with a thin layer of gold for 30–60 seconds, the freeze-dried CNFs were scanned by a scanning electron microscopy (S-4800, Hitachi High-Tech., Japan) at an accelerating voltage of 20 kV.

### Thermogravimetric analysis (TGA)

TGA was performed to evaluate degradation characteristics of the untreated samples and the obtained CNFs with a Thermogravimetric Analyzer (Netzsch 209F1, Germany) from room temperature to 600°C with a heating rate of 10°C/min under N_2_ (30 ml/min). A sample of 6 mg was used for each run.

## Results and Discussion

### Chemical composition

The content of *α*-cellulose and hemicellulose of the obtained chemical treated fibers (CHFs) is 95.71% and 4.29%, respectively. However, the content of cellulose and hemicellulose in coconut palm petiole fiber is 33.29% and 33.61%, which confirms that non-cellulosic compounds such as wax, pectin and lignin have been removed effectively with chemical treatments.

During the experiment of chemical treatments, the cellulose yield left is 32% as comparing to the initial mass of the woody powder sample. That is to say, there is 1.29% mess loss of cellulose. Thatis probably caused by the washing operation of the treated insoluble substanceswith deionized waterinto neutral in every step of isolating nanocellulose from the woody powder.

### Fourier Transform-infra Red Spectroscopy (FTIR) analysis

**Fig 2:**
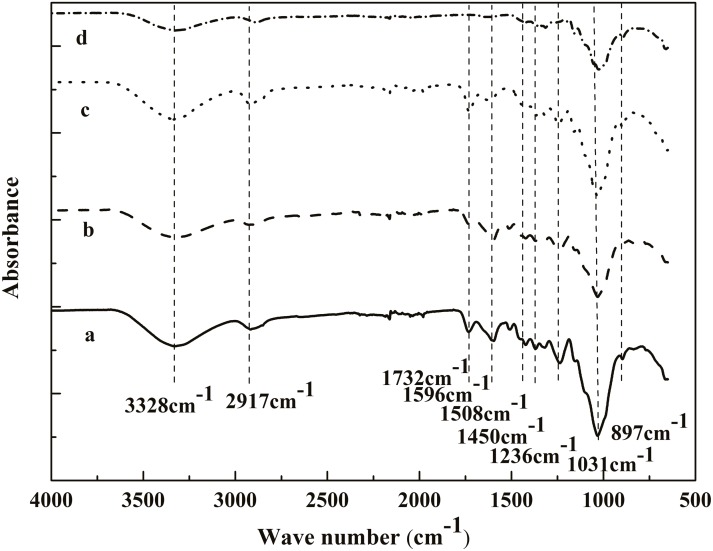
FTIR spectra of fibers at different chemical treatment stages: (a) untreated, (b) benzene/ethanol treated, (c) acidified sodium chlorite treated, and (d) sodium hydroxide treated.

Fig 2 shows the FTIR spectra of the untreated, benzene-ethanoltreated, acidified sodium chloritetreated and sodium hydroxidetreatedcoconut palm petiole fibersamples, and the main absorption peaks are listed in Table 2.

**Table 2:** Main absorption peaks of the samples at different treating stages.

a	b	c	d	Involved groups
3328	3323	3331	3332	O-H stretching
2917	2915	2924	2924	C-H stretching
1732	1730	1730	-	Carboxyl groups(C = O) in the acids and esters of acetic, *p*-coumeric, ferulic, and uronic acids
1508	1508	-	-	Aromatic C = C stretching in lignin
1236	1233	1234	-	C-H, O-H or CH_2_bending frequencies
1031	1030	1032	1031	C-O, C-C stretching or C-OH bending
897	897	897	897	C-O stretching and C-H vibration in cellulose

The dominant peaks in the region between 3600 cm^-1^ and 2800 cm^-1^, which are observed in the spectra of all samples, are due to the stretching of functional groups of-CH and-OH, and the band at 897cm^-1^ isassociated with the C-H rocking vibrations of cellulose[22, 23]. These results indicate that the cellulose component has not been removedby the chemical treatments. The sharp peak at 1732cm^-1^ in the spectra of the untreated, benzene-ethanoltreated, acidified sodium chloritetreated samples refers to the carboxyl groups in acids and esters ofacetic, *p*-coumeric, ferulic, as well as uronic acids, which are main constituents of benzene-ethanolextractivesand hemicellulose [22,24]. It was found that the intensity of this band decreased in the spectra of the benzene-ethanoltreatedsample (Fig 2b), demonstrating the partial removal of the extractives after benzene-ethanol extraction.And in the spectra of the sodium hydroxidetreatedsample (Fig 2d), the band almost vanished, due to the removal of most non-cellulosic materials.Thepeak at 1508cm^-1^, which is associated with aromatic skeleton vibration of aromatic rings of lignin[24,25], also disappears in the spectra of the acidified sodium chlorite treated sample (Fig 2c), showing the removal of lignin from the sample.The intense bands at1236cm^-1^ and 1031cm^-1^ correspond to-C-H,-O-H or-CH_2_bending frequenciesand C-O, C-C stretching or C-OH bending of cellulose and hemicellulose, respectively[22, 24, 25]. Their intensity significantly decreases after sodium hydroxide treatment (Fig 2d), revealing the most removal of hemicellulose. This result is supported by the data of the content of *α*-cellulose and hemicellulose in the obtained CNFs in this study.

### X-ray Diffraction Technique (XRD) analysis

**Fig 3:**
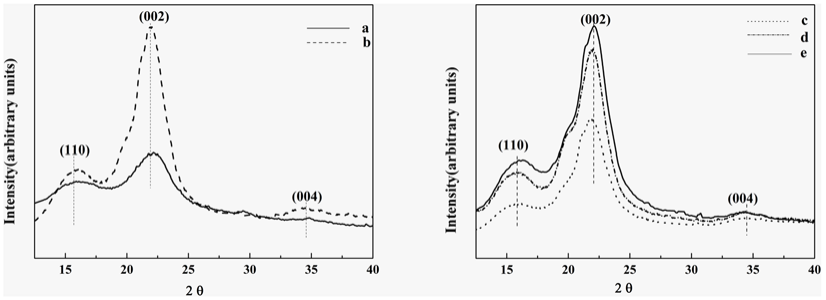
X-ray diffraction patterns of (a) untreated, (b) sodium hydroxidetreated, (c) CNF-1, (d) CNF-2 and (e) CNF-3.

Cellulose has a well prominent crystalline structure due to its hydrogen bonding and Van der Waals forces existing between adjacent cellulose molecules contrary to hemicellulose and lignin, which are amorphous innature[15].The increase in crystallinity of the cellulose fiber is expected to increase their rigidity and stiffness;higher crystallinity is associated with higher tensile strength [26].X-ray diffractogram is used to investigate the effect of chemical and mechanical treatmentson the crystallinity and crystal type of the cellulose fiber. The X-ray diffraction patterns of the untreated sample, chemical pretreated sample, CNF-1, CNF-2 and CNF-3 are shown in Fig 3. All patterns present three peaks around 2θ = 16°, 22° and 34°corresponding to the (110), (002) and (004) planes, which are well defined crystalline peaks of cellulose *I*.The crystallinity of the samples is summarized in Table 3.Compared to the untreated sample, the sample with successive chemical treatments has higher values of Icr. This is due to the partial removal of amorphous phases like hemicellulose and lignin by chemical treatments.Fengelet al. believed that the crystalline region couldresist theattack of dilute acid, but the amorphous parts could be destroyed during acid treatment[27]. Lima et al. deemed that hydronium ions could penetrate into accessible amorphous regions of cellulose upon acid treatment and allowed hydrolytic cleavage of glycosidicbonds,which eventually released individual crystallites [28].On the other hand,the alkali treatment could increase stiffness of the fiber as the impurities present in the fiber were removed during the treatment[29].

**Table 3:** Crystallinity index (Icr) of coconut palm petiole fibers at different stage of treatment.

Sample	Icr (%)
Untreated	38.00
Chemical pretreated	70.36
CNF-1	71.00
CNF-2	71.20
CNF-3	67.20

In Table 3, the cellulosecrystallinity of CNFs also shows a slight increasingtrend with the increasing passing times up to 15. Ordered arrangement of the crystalline cellulose in the structure during the grinding operation might take place which increased the crystallinity [30]. Chirayila et al. [5] and Li et al. [31] also reported that the realignment of monocrystals of cellulose during mechanical treatment might occur in parallel and thus improvedits crystallinity. However, further increasing passing times to 20, the cellulose crystallinity decreases. This might caused by degradation of the CNFs due to the continuous shearing force generated by the grinding disks [32].

### Scanning Electron Microscopy (SEM) analysis

**Fig 4:**
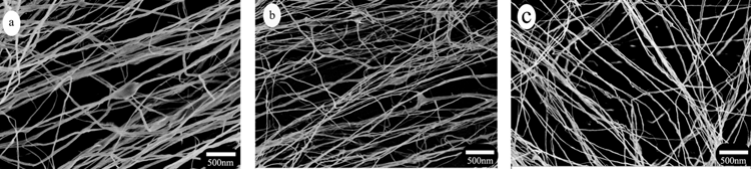
FE-SEM images of (a) CNF-1, (b) CNF-2, and (c) CNF-3.

SEM images of the obtained CNFs with different grinding passing times are shown in Fig 4. It’s clear that the diameters of CNFs decreasewith the increasing grinding passing times from 10 to 20. The grinding treatment gradually peels off the external cell wall layers (*P* and *S*_*1*_layers) with the exposed *S*_*2*_ layer, resulting inthe internal fibrillation loosening in the fiber wall, and as a result more fibrils are separated from the fibril bundles [33]. For CNF-1, passing through the grinder 10 times results in an average length of 18um, a broad diameter distribution of 50–120nm, and an aspect ratio of 150–360. As the grinding times increase to 15 (CNF-2) and 20 (CNF-3), the diameter of CNFs telescopes into 25–50nm and 25–40nm, and the aspect ratio turns into 320–640 and 375–600, respectively. The aspect ratio of CNFs isolated from coconut palm petiole in this study is much higher than that of other resources, such as coconut husk (60)[17], wheat straw (90–100) [22] and corn cobs(53)[12]. This result indicates that coconut palm petiole CNFs could be an ideal reinforcement material for composites due to its high aspect ratio [5, 24].

### Thermogravimetric analysis (TGA)

**Fig 5:**
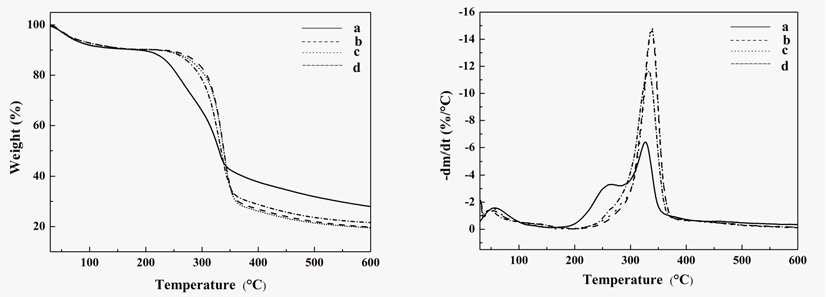
TG and DTG curves of (a) untreated, (b) CNF-1, (c) CNF-2, and (d)CNF-3.

Thermogravimetric analysis and differentialthermogravimetry (DTG) of the untreated coconut palm petiolefiber and the isolated CNFs are shown in Fig 5, and the related data are summarized in Table 4.

**Table 4:** Degradation characteristics of the coconut palm petiole fibers.

Coconut palm petiole fibers	T_i_ (°C)*	T_max_(°C)**	Residue after 550°C(%)
Raw	186	326.61	31.66
CNF-1	253	337.81	21.93
CNF-2	253	341.03	21.20
CNF-3	253	333.05	23.71

*T_i_ is the initial decomposition temperature

**T_max_ is the temperature with maxmium decomposition rate

In all samples, the weight loss of cellulose is divided into three steps. A small weight loss in the region 35–130°C is mainly due to the vaporization and removal of boundwater in the cellulose[34], and depends on initial moisture content of the fiber. The weight loss at temperature around 228–315°C is attributed tothermal depolymerization of hemicellulose and cleavage of glycosidic linkagesof the cellulose [35].When increasing temperature to 346–402°C, there is another weight loss because of the degradation of lignin and cellulose. The fiber residue remaining after heating to 550°C indicates the presence of carbonaceous materials in the coconut palm petiole fiber in the nitrogen atmosphere [22]. Compared to that of the CNFs,the amountof residueat high temperature for the untreated coconut palm petiole fiber is increased due to the presence of ash as well as lignin[36].

As shown in Fig 5, the initial degradation temperature (T_i_) and maximum degradation temperature (T_max_) of the untreated fiber are 186°C and 326.61°C, respectively;while for CNFs, the on-set degradation takes place at 253°C and the maximum degradation temperature are 337.81°C (CNF-1), 341.03°C(CNF-2) and 333.05°C (CNF-3), respectively.It demonstrates that the chemical-mechanicaltreatments improve the thermal stability of fibers, and this result is accorded withAlemdar et al. [22] and Maheswariet al[37].This improvement of thermal stability is ascribed to the removal of hemicelluloses, lignin and pectin in the CNFs, which have a lower decomposition temperature compared to cellulose[38]. However, the maximum degradation temperature (T_max_) increases first but decreases upon 20 passes through the grinder. This may be explained severemechanical treatments destroy the crystallline of cellulose. It can be concluded that the better thermal stability has been related to partial removal of hemicellulose and lignin and higher crystallinity of the cellulose [39].These results are very consistent with the result obtained from XRD.

## Conclusions

The present work showed that high quaility CNFs can be successfully isolated from coconut palm petiole residues.FTIR and XRD showed that chemical treatments removed all lignin and most of the hemicellulose and increased the crystallinity from 38.00% to 70.36%. With the grinding time increased from 10 to 15, the crystallinity kept slightly growing. However, the decrease of crystallinity occurred when the grinding continue increasing to 20. The SEM images showed that grinding passing times affected the diameter and the aspect ratio of CNFs greatly, and a conclusion was drawn that high quality of CNFs could be isolated from coconut palm petioleresidues with chemical treatments in combination of 15times of grinding followed by 10 times of homogenization. The results of TGA-DTG revealed that the chemical-mechanical treatments improved the thermal stability of fibers, and the CNFsisolated from 15 times grinding passinghad the best thermal stability.
